# Molecular Dynamics Simulations of Self-Assembled E_2_(SW)_6_E_2_ Peptide Nanofibers: Implications for Drug Delivery and Biomimetic Material Design

**DOI:** 10.1021/acsphyschemau.5c00028

**Published:** 2025-05-08

**Authors:** Karinna Mendanha, Guilherme Colherinhas

**Affiliations:** Instituto de Física, 67824 Universidade Federal de Goiás, Goiânia, Goiás 74690-900, Brazil

**Keywords:** molecular dynamics, nanotapes, hydrogen bond dynamics, peptide self-assembly

## Abstract

This work investigates the molecular dynamics of the peptide nanofiber E_2_(SW)_6_E_2_, a biomolecule/structure in an aqueous solution, characterized by hydrophilic and hydrophobic contrasts. Through classical molecular dynamics simulations, the study examines the energetic, structural, and dynamic properties of this nanofiber, with a focus on energetic and hydrogen bond (HB) interactions between peptides and peptide-water. Simulations of different fiber lengths indicate that larger models exhibit increased structural stability and longer HB lifetimes, contributing to enhanced fiber flexibility and integrity. Additionally, the analysis of the mass density profile along the nanofiber length reveals local decreases (but not zero) in mass density. The results further emphasize the potential of these structures for applications in ion and drug transport due to their hydrophobic core and hydrophilic surface. This work provides a comprehensive understanding of molecular interactions in self-assembled bionanomaterials in aqueous solutions.

## Graphical abstract

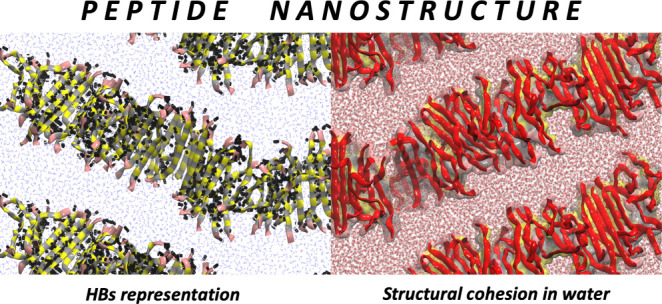

## 1 Introduction

Although experimental results highlight certain structural features of peptide self-assembly, there is a growing demand for structural characterization at the molecular level, achieved through computational simulations with high methodological and modeling precision. Recent studies involving molecular dynamics (MD) have shown that this technique provides reliable results when compared to experimental data and contributes to the understanding of the formation of such nanostructures.^1–9^ Previous theoretical results have also indicated that peptides forming membranes, sheets, or nanofibers, which contain polar amino acids in their structure, ensure a cohesive and strongly aligned organization due to the formation of a network of hydrogen bonds (HB).^10–12^ Recent studies also show that the dimensions of the system simulated using classical MD with periodic boundary conditions (PBC) interfere with some properties of the system,^13–15^ and depending on the objective of the study, these effects should be considered. In this sense, some results highlight those simulations using small surface areas for the peptide membrane, for example, may result in nonconverged values for the system properties, which are only observed in regions simulated under certain conditions where the PBCs do not affect the region.^15^ For peptide fibers, studies show that the simulated fiber length is crucial to observe their undulations and formation.^12,16^ In both cases, HBs are the predominant property linked to the cohesion and formation of the nanostructure, being strongly affected by poor system modeling.^15,17^

Parallelly, there are a series of studies that highlight applications and properties of self-assembling peptide materials.^9,18–24^ Among these, there is a class of peptides known as multidomain peptides (MDP), which are a class of self-assembling peptides organized in a β-sheet motif, resulting in a nanofibrous architecture. This structure is stabilized by hydrophobic packing in the fiber core and a HB network along the fiber’s long axis. In this context, there is a class of peptides of the type Ψ_n_(ΘΩ)_m_Ψ_n_, with Ψ = glutamic acid (Glu;E) or lysine (Lys;K), Θ = glutamine (Gln;Q), serine (Ser;S), threonine (Thr;T) or cysteine (Cys;C), Ω = leucine (Leu;L), phenylalanine (Phe;F), tyrosine (Tyr;Y) or tryptophan (Trp;W), n = 1–4 and m = 6–8, which are specifically designed for laminar fiber organization, fundamentally forming structures with hydrophilic or hydrophobic faces depending on how the peptide is stacked during self-assembly. Structures such as K_2_(SL)_6_K_2_ favor drug transport by diffusion, while a slight deformation in the peptide’s formation, K_2_(SL)_2_SA­(SL)_3_K_2_, promotes the formation of a pore that aids in drug transport via intrafibrillar encapsulation.^20^ In fact, what contributes to drug transport through these materials is related to the affinity between the drug and the peptide. However, the characteristics of these MDPs provide a material that can exhibit high hydrophilic affinity in one region and be completely hydrophobic in another, similar to what is currently observed in lipopeptides.^25–29^

The sequence E_2_(SW)_6_E_2_ was chosen for its suitability for self-organization into stable nanofibrils and its ideal intrinsic characteristics for drug delivery applications. The solid hydrophobic core formed by the stacking of tryptophan (W) residues provides cavities that favor the encapsulation of aromatic and lipophilic molecules via π – π interactions, while the hydrophilic crown of serine (S) residues and the charged glutamate (E) termini confer high aqueous solubility, colloidal stability, and the potential for controlled release in response to pH variations. The interactions between tryptophan residues play a crucial role in the hydrophobic region, as the stacking of indole rings promotes aggregation in aqueous solution. Furthermore, the hydrogen bonds formed on the surface enhance interaction with the biological milieu, improving biocompatibility and reducing unwanted aggregation. This combination of encapsulation selectivity, structural stability, and environmental responsiveness makes E_2_(SW)_6_E_2_ a promising nanovehicle for drug transport and release in biological systems. In this context, computational modeling of peptide nanomaterials becomes a fundamental tool for understanding their properties, especially those that are difficult to obtain experimentally with current techniques. Classical MD simulations of the E_2_(SW)_6_E_2_ MDP were used to evaluate its energetic, dynamic, and structural characteristics, including the dynamics of its hydrogen bonds (HBs). This approach allowed detailed analyses, including lifetimes, rupture energies of HBs, and the extraction of information that is inaccessible through experimental methods, using advanced autocorrelation techniques.

## 2 Methodology

Initially, a peptide was constructed using the PyMOL program^30^ from the amino acids glutamic acid (E), serine (S), and tryptophan (W), following the sequence E_2_(SW)_6_E_2_, as shown in Figure 1a. It is important to highlight that the alternating structure, between S and W, that composes the body of the peptide presents a hydrophobic region (composed only with W residue) and a hydrophilic region (composed only with S residue). Thus, based on the principles described by Moore and Hartgerink,^20^ the alternating pattern of hydrophobic and hydrophilic residues in the peptide core, along with the charged terminals (E and K), was used to construct the base structure of the peptide that makes up the nanofiber. This organization promotes greater solubility, structural stability, and controlled aggregation of the nanofiber, and this design approach is aligned with the formation of specific nanostructures, enabling the understanding of the self-organizing properties of the proposed structure. This sequence of amino acids that make up the E_2_(SW)_6_E_2_ peptide presents, in addition to the central hydrophobic/hydrophilic region, a concentration of charged regions at its ends, making the peptide with a net electric charge equal to – 4*e* which is distributed in the E residues, while the S and W residues are neutral. From this peptide, a dimer was created with the best possible fit (Figure 1c), and then this dimer was replicated 10 times in the *z*-direction to build the ribbon, as shown in Figure 1d. The composed structure therefore presents a hydrophobic region in its interior, where the structure favors π-π interactions between the cyclic chains of the W residue, while the external region is abundant in S residues, which enhance strong hydrogen bond interactions with water molecules. The amino acid glutamic acid is located at the left and right edges of the peptide ribbon. Due to the overall negative charge, the simulation box was initially constructed with the ribbon and positive ions (potassium) to ensure that the electrical charge in the system is neutral. Due to the twisting of the peptide (Figure 1b), the fitting between the two peptides that make up the dimer has an offset, which can be observed in the complete structure (Figure 1d). Despite this offset of the dimer and the twisting of the peptide, the fitting between each pair of peptides replicated in the z direction favors HBs along this direction, thus constructing a network of HBs that will keep the structure cohesive during the MD simulation. Thus, the construction of the initial structures was designed considering the profile of the E_2_(SW)_6_E_2_ peptide and its possible directed interactions, such as the formation of β-sheets and hydrophobic interactions that contribute to system packing. Although it is possible to randomly distribute peptides in a simulation box, such an approach requires extremely long and impractical MD simulations, both in terms of computational time and data analysis, to observe complete self-organization. Thus, the systems were simulated from preorganized structures, followed by thermalization steps involving peptides, ions, and solvent, allowing the system to achieve an organization that characterizes the self-assembly process. This method enables the evaluation of the stability and structural cohesion of the fibers on larger scales while maintaining a good balance of computational cost.

**Figure 1:**
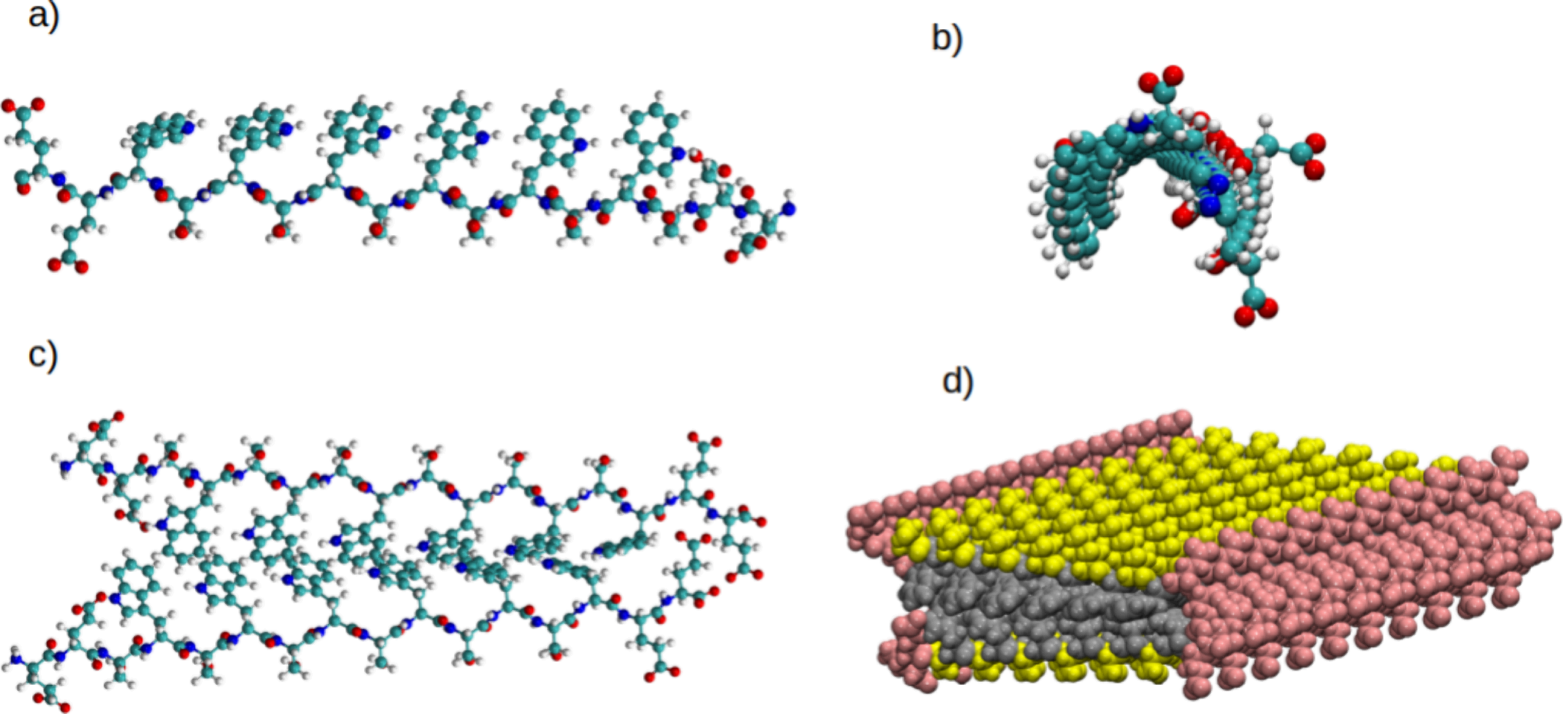
Structural representation of the E_2_(SW)_6_E_2_ peptide: (a) Side view representation of the peptide highlighting its composition along the backbone; (b) Representation of the peptide emphasizing its twist along the backbone; (c) Representation of the juxtaposition of two peptides forming the main dimer for nanofiber assembly, with a focus on the central region featuring hydrophobic interactions composed of stacked rings; and (d) Representation (in vdW) highlighting the replication of the dimer along the length of the nanofiber. For (a)-(c): Green represents carbon atoms, white represents hydrogen atoms, red represents oxygen atoms, and blue represents nitrogen atoms. For (d): Pink represents glutamate residues (E), yellow represents serine residues (S), and gray represents tryptophan amino acids (W).

Several strategies were used to model this system, but here we will present only the successful approach (a comment on the strategies that were not successful is provided in the Supporting Information). First, the peptide nanostructure shown in Figure 1d was placed in a rectangular box with dimensions described in Table 01, where the *z*-axis coincides with the length of the nanofiber. Potassium ions were randomly inserted into the same simulation box, and this system was subjected to short MD simulations, alternating between NPT and NVT ensembles, until a cohesive structure was obtained. The objective of this strategy is to gradually adjust the length of the nanofiber as the ions are adsorbed by the structure. This process continues until a cohesive structure formed by the ions and peptides is achieved. This cohesive structure was the starting point for four additional simulation boxes that replicate the original system. The first box (Model-01) is the original box with a structure formed by 20 peptides and 80 K^+^ ions; the second box (Model-02) was obtained by replicating Model-01 along the direction of the laminar fiber and contains 40 peptides and 160 K^+^ ions. Models 03, 04, and 05 were obtained following the same logic, and their compositions are shown in Table 01. The models studied in this work are presented in Figure 2. After the structuring phase of the peptide laminar fiber, all systems were solvated using the Gromacs program.^31^ From these new boxes containing the solvated structures, the systems were subjected to classical MD simulations to achieve the thermodynamic equilibrium necessary for the production phase, where statistical analysis is performed. Thus, each Model-XX underwent two stages: one aimed at achieving thermodynamic equilibrium of the structure in solution, which, in total, involved ∼ 15 ns of alternating MD simulations in the NPT and NVT ensembles (this step is completed when parameters such as pressure, temperature, total potential energy, Coulomb and Lennard-Jones interaction energy, simulation box dimensions, and even the mass density profile remain converged); and a final MD simulation stage aimed at producing a long classical trajectory of the Model-XX systems for statistical analysis.

**Figure 2:**
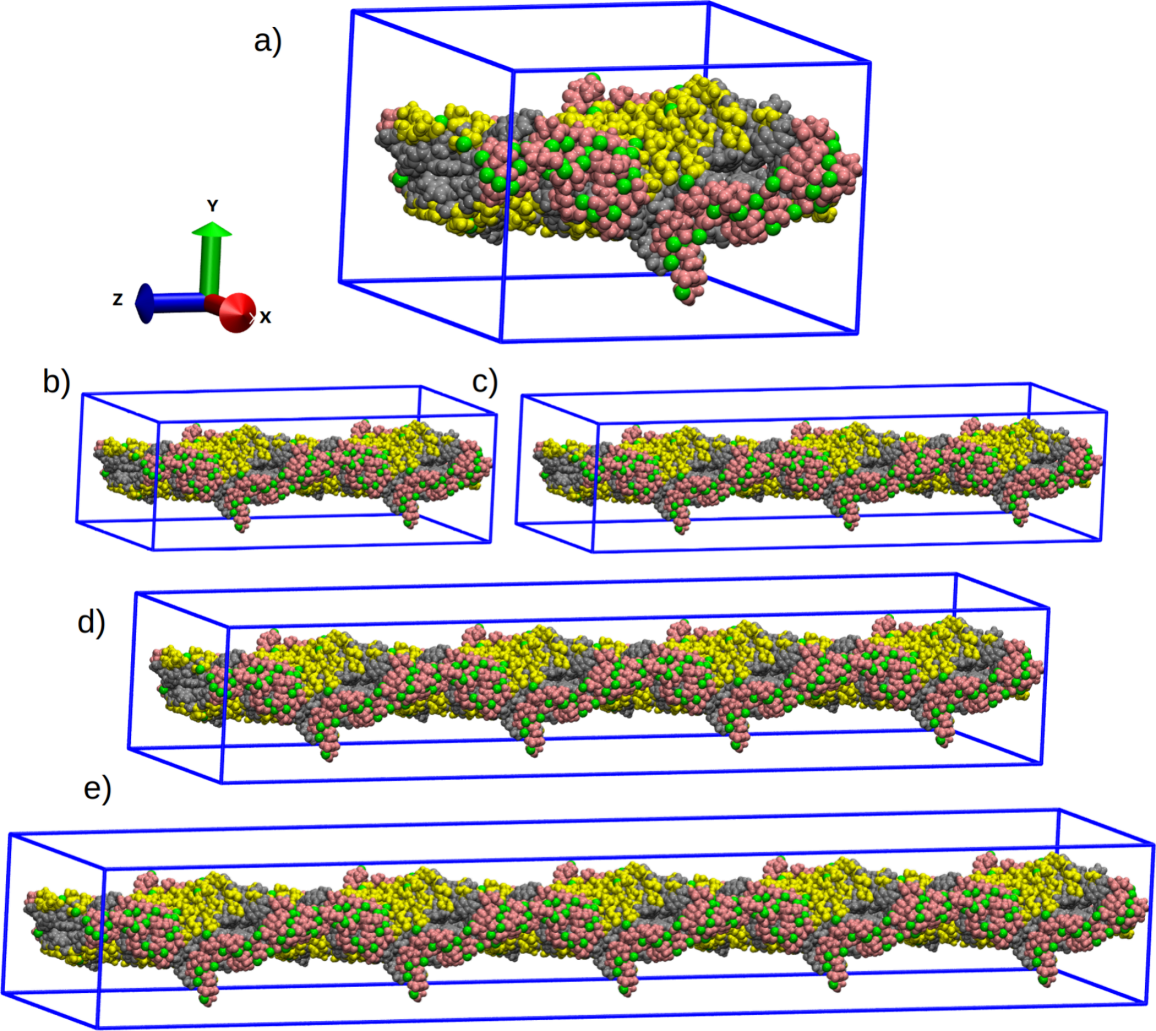
Representation of the initial configuration of the simulated laminar nanofibers in this work. The *Z*-axis represents the dimension of the simulation box associated with the nanofiber length, which is linked to the periodicity of the structure. Water molecules were omitted for better visualization. (a) Model-01; (b) Model-02; (c) Model-03; (d) Model-04; and (e) Model-05. Pink represents glutamic acid (E) residues, yellow represents serine (S) residues, gray represents tryptophan (W) amino acids, and green represents potassium ions (K^+1^).

**Table 01:** Total Number of Molecules/Ions Present in Each Model Simulated in This Work^a^

	Model-01	Model-02	Model-03	Model-04	Model-05
# Peptides	20	40	60	80	100
# Water	6,321	12,642	18,963	25,284	31,605
# K^+^	80	160	240	320	400
# atoms	24,503	49,048	73,605	98,045	122,617
x	6.79	6.79	6.79	6.79	6.79
y	4.85	4.85	4.85	4.85	4.85
z	7.25	14.61	21.70	28.60	36.12

a We also present the total number of atoms that compose each simulation box. The values expressed by x, y, and z (in nm) correspond to the dimensions of the simulation box of each model. The value for z also corresponds to the length of the simulated nanofiber.

The final MD simulation stage consisted of 100 ns of computational simulation. At this point, (a) the system started the production phase already in a relaxed state and thermodynamic equilibrium, having reached its most favorable and stable configuration during the preproduction phase, and (b) the simulation was performed under the NVT ensemble due to the challenges in controlling pressure during computational simulations of this type of peptide structure, as highlighted in the reference.^12^ Pressure control in MD simulations of peptide fibers presents unique challenges due to their geometry and the critical interactions that stabilize the system, such as HBs, which are highly sensitive to peptide distance, angle, and alignment, potentially compromising the modeling of the system if pressure along the fiber is erroneously applied. Furthermore, the anisotropic nature of the pressure distribution—differing between the xy-plane and the *z*-axis due to periodic boundary conditions and their anisotropic application—requires careful adjustments to the simulation box to avoid distortions that could compromise structural stability. For this reason, NPT simulations were performed in short intervals, interspersed with NVT simulations to accommodate the nanostructure within the simulation box. This alternating simulation process converges toward thermodynamic equilibrium, enabling the formation of a fiber-like nanostructure that reproduces experimental characteristics. Thus, in this study, a detailed and carefully adjusted modeling approach was employed to replicate experimental conditions, prioritizing the maintenance of the structural integrity of the peptide nanofibers, as demonstrated in previous works.^2,12,17^

After the production phase, we obtained a trajectory containing 50k configurations for the statistical analysis of the properties to be presented. In all our simulations, each integration of the MD motion equations was performed with a time step of 1 fs. To calculate the electric potential, the Particle-Mesh Ewald (PME) method^32^ was employed with a cutoff radius of 1.2 nm. For van der Waals energies, the Potential-Shift Verlet method was used, also with a cutoff radius of 1.2 nm. Figure S1 in the supporting material highlights the potential energy behavior of all systems during the production stage. In the simulations where pressure was maintained constant, a pressure of 1.013 bar was sustained using semi-isotropic Parrinello–Rahman coupling,^32,33^ with adjustments every 4 ps and a compressibility of 4.5 × 10^–5^ bar^–1^. To keep the temperature constant at 300 K, the v-rescale algorithm^34^ was applied every 0.1 ps. Figure S2 in the supporting material highlights the temperature behavior of all systems during the production stage. The LINCS algorithm^35^ was used to constrain bond lengths and ensure the stability of the simulated structure during the MD. The force field used to model the entire system was CHARMM36,^36–38^ while TIP3P^39,40^ was employed to model the water molecules. The images were obtained using the VMD program,^41^ and the analyses were performed using the Gromacs software^42^ package and we used the Packmol program^43^ to add the ions to the simulation box. For our results, we will perform an energetic and structural analysis to observe the influence of system size. We will analyze the structure and dynamics of HBs from van der Spoel and Luzar-Chandler theory^44–46^ and the Ramachandran plot.^47^ For the identification of HBs, we adopted the geometric criteria commonly used in the literature: a maximum distance of 0.35 nm between the donor (D) and acceptor (A) atoms, and a *D* – *H*···*A* angle of less than 30°. These parameters were applied consistently in the analyses of the average number of HBs, HBs-lifetime, and rupture energy, following the approach proposed by Luzar–Chandler theory. To ensure that thermodynamic equilibrium was achieved in the simulated systems, we monitored the stability of physical parameters during the production phase. The convergence of these parameters was confirmed throughout the entire production phase. The corresponding curves for these properties are presented in the Supporting Information.

Given the increasing number of peptides (from 20 to 100), the computational cost of the simulations also increased accordingly. The simulations were run on high-performance computing clusters, utilizing parallelization techniques to distribute the computational load across multiple cores, significantly reducing execution times compared to sequential simulations. For larger systems, with up to 100 peptides, the production phase took approximately 30 days on clusters with 16 cores, while simulations of smaller systems were completed in about 5 days. Additionally, property calculations, particularly for hydrogen bond (HB) analyses, were computationally intensive. Each pairwise HB analysis (e.g., peptide-water interactions) in the larger models required 15–20 days and demanded up to 128GB of memory. Parallelization was primarily applied to long-range interaction calculations, such as the PME method for electrostatic interactions, which ensured efficient performance even for larger models. These computational strategies and time estimates provide useful guidance for research groups looking to replicate or expand on the models presented in this study.

## 3 Results

### 3.1 Structural Analyses

The mass density profile is an important analysis in MD simulations of laminar structures, fibers, membranes, and others, allowing us to understand the mass distribution in relation to a spatial coordinate. In this case, Figure 3 shows the mass density profile along the *z*-axis, which corresponds to the length axis of the fiber simulated in this work. In these graphs, the projection of the mass onto the *z*-axis shows how much of the fiber is present at a given position on the *z*-axis. We can observe that, despite some variations, the mass is distributed along the simulation box, reaching over 400 kg/m^3^ in certain regions. Another observation from the mass density profile is that the fibers do not break along their structure, maintaining the fiber shape throughout the simulation. The average mass density of the five models studied is around 300 kg/m^3^, and it is also noticeable that there is periodicity in the mass distribution of the structure. We also observe that, for most of the simulation time, the models remain above the average of 300 kg/m^3^, indicating that there are regions where the fiber may fold or organize in such a way that some regions are more (or less) filled.

**Figure 3:**
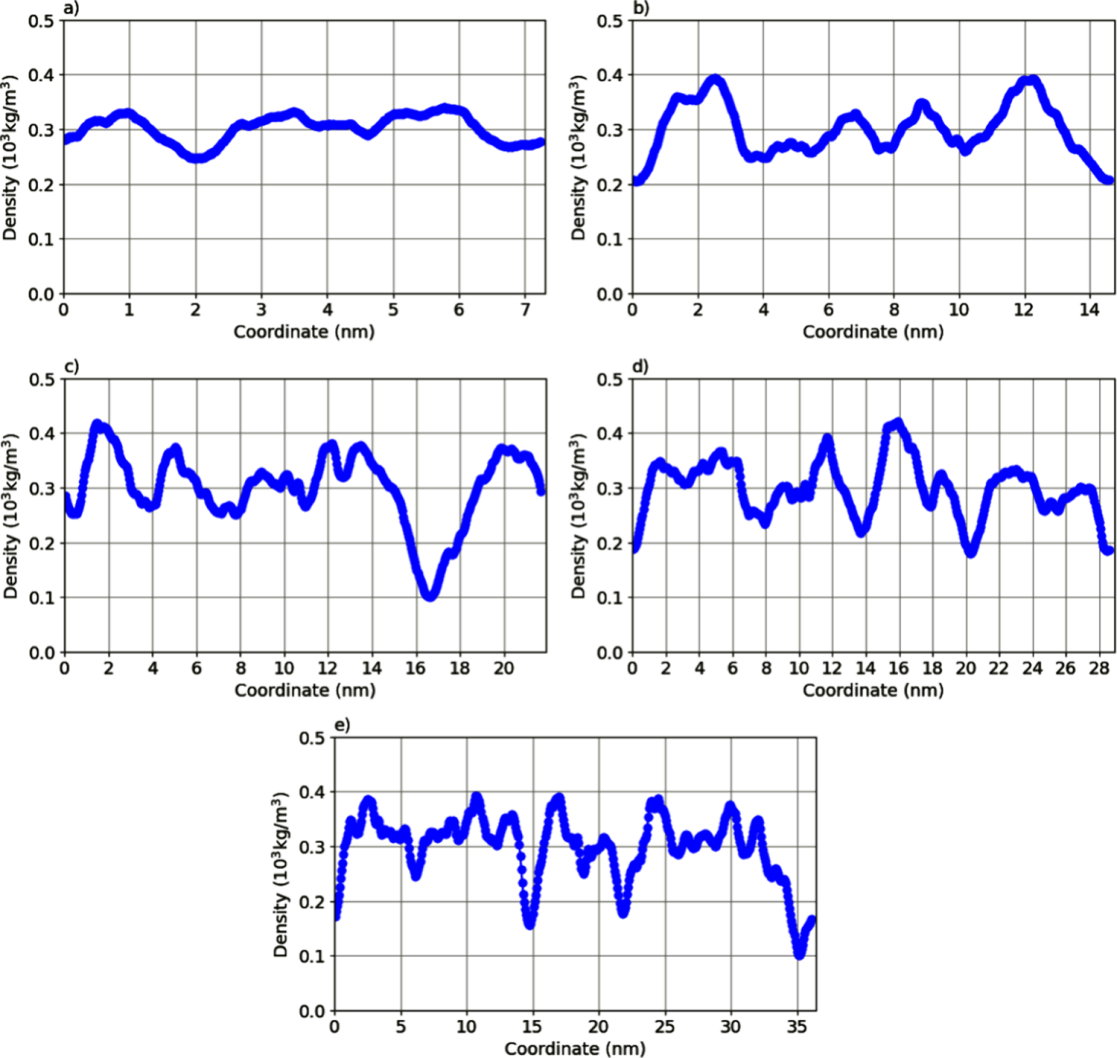
Analysis of the mass density profile along the *Z*-axis for peptide structures. The simulation box is divided into small slices along the Z direction. In each slice, the mass of the peptides is calculated and recorded as a projection along the *Z*-axis, which corresponds to the length of the nanofiber. Note the periodicity at the edges in the mass density profile, representing the continuity of the nanofiber. (a) Model-01; (b) Model-02; (c) Model-03; (d) Model-04; and (e) Model-05.

We should also emphasize that, although most of the nanofiber shows a cohesive mass density and structure, there are regions that appear less populated (lower mass projection on the *z*-axis), which may indicate a rupture in the fiber structure. This is particularly seen in larger models (Model-4 and Model-5), suggesting that simulations with smaller structures (such as Model-01 or 02) may mask the correct physical form of the peptide nanostructure. This can also be observed in Figure 4, which represents a configuration of the Models-XXs and their replicas due to the periodic boundary conditions (PBCs). Only with this type of visualization can we observe that larger models (04 and 05), which show a discontinuity in the structure within the box, actually demonstrate an almost perfect continuity when the PBCs are considered. It is also worth noting that smaller structures are unable to display the undulations these structures may undergo, as is the case with Models-04 and 05. As we can see from the results presented, the assessment of structural maintenance can be performed using the mass density profile, which provides an average of the configurations obtained during the MD simulations. Additionally, while there are areas of potential rupture in the nanofibers (observed in some configurations in Figure 4), this also reflects an experimentally observed behavior indicating that these structures have variable lengths. The results presented here show that the mass density in the rupture areas may exhibit a reduction greater than 50% of the local mass of the nanofiber, but importantly, there is no decrease to zero mass in the statistics shown in Figure 3, which would indicate a permanent rupture throughout the entire MD simulation. We believe that this nanofiber rupture phenomenon in solution can also be interpreted as a dynamic process.

**Figure 4:**
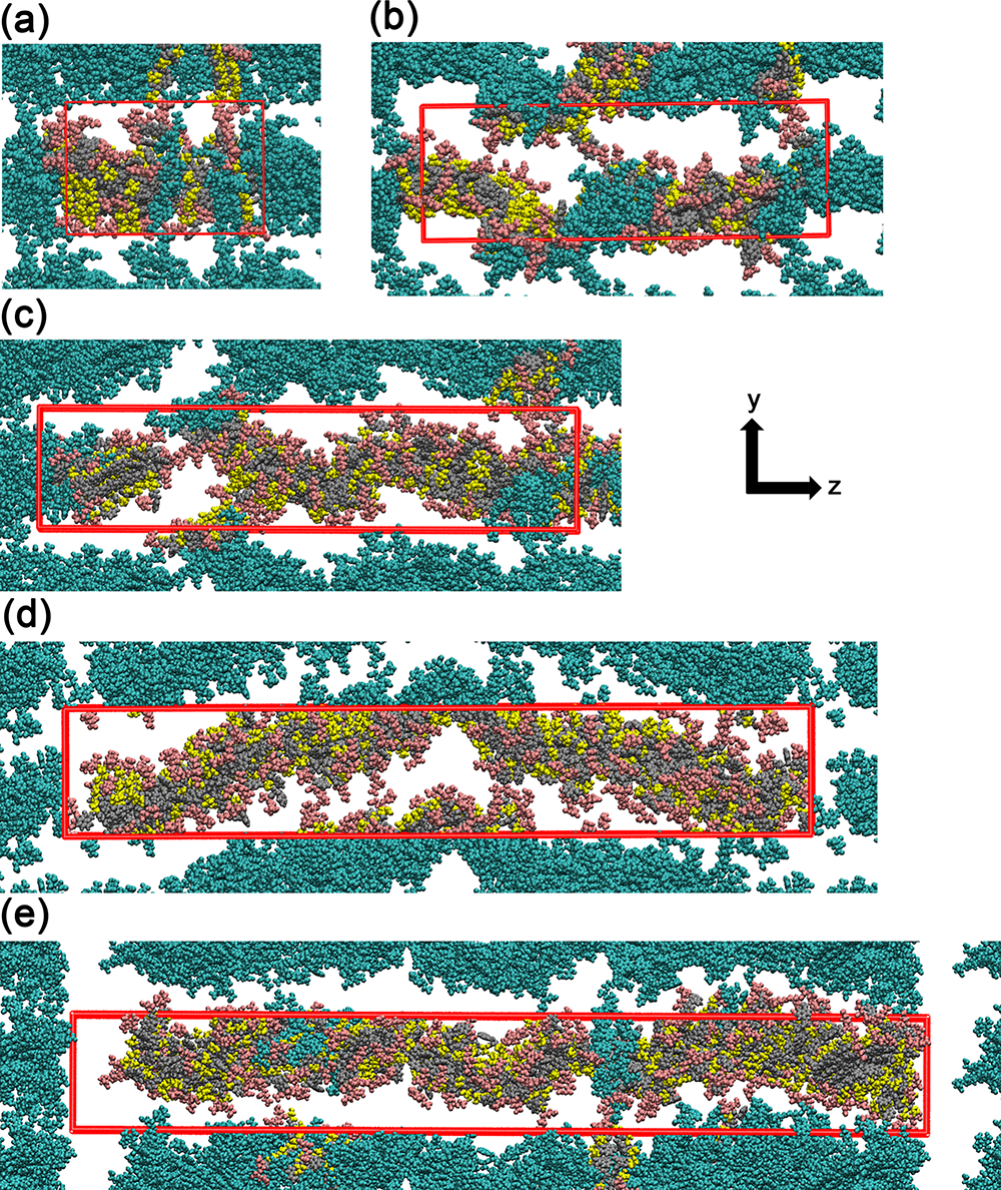
Representative snapshots of peptide nanofiber structure configurations in the YZ-plane, extracted from the production-stage trajectory. Peptides are colored by residue type—pink for Glu, yellow for Ser, and gray for Trp. The Trp residues (gray) form π – π interactions through stacking of their indole rings, particularly between adjacent Trp residues along the nanofiber structure axis, which contribute to the stability of the hydrophobic core. These interactions are crucial for nanofiber assembly and aggregation in aqueous solutions. Ser residues (yellow) form hydrogen bonds with the solvent, enhancing the hydrophilic surface stability, while Glu residues (pink) are oriented at the nanofiber structure ends, contributing to the overall structural cohesion. Cyan structures show periodic replicas to illustrate nanofiber continuity across the simulation box. Panels (a)–(e) correspond to Model-01 through Model-05, respectively, highlighting how increasing nanofiber structure length enhances backbone undulations while preserving the integrity of the hydrogen-bond network along the *Z*-axis.

Additionally, the radius of gyration is the root-mean-square distance of each atom in a protein from the center of mass of the nanostructure, serving as a quantitative measure of the nanostructure (nanofiber) size. In the present study, the values of the radius of gyration (*R*_*g*_) and the end-to-end distance (*R*_*ee*_) show a progressive increase across the studied models. The results highlight the influence of nanofiber size on its structural conformation, as a comparison reveals that *R*_*g*_ values range from 4.6 (Model-02) to 10.7 (Model-05), representing an increase of ∼ 131%, while *R*_*ee*_ increases from 12.9 to 37.4 (an increase of ∼ 190%). This suggests that as the nanofiber grows, its end-to-end extension expands more significantly than its dispersion around the center of mass, indicating a greater structural elongation rather than a simple isotropic expansion. Finally, the *R*_*ee*_/*R*_*g*_ ratio increases as the model grows longitudinally, varying from 2.8 (Model-02) to 3.5 (Model-05), further reinforcing the hypothesis that larger nanofibers tend to adopt more elongated conformations.

Due to the structural characteristics of the nanofiber, it was possible to estimate the volume of the peptide structure. For Model-1, the estimated volume was ∼ 56 nm^3^, for Model-2 it was ∼ 110 nm^3^, Model-3 indicates ∼ 166 nm^3^, Model-4 shows a volume of ∼ 224 nm^3^, while Model-5 shows a volume of ∼ 280 nm^3^. Based on this parameter, we can also estimate the number of peptides per unit volume, which fluctuates around 0.4 peptides/nm^3^ in all models. The solvent-accessible surface area (SASA) was obtained using the Solvent Accessible Surface Area calculation method, which considers the interaction between the molecule and a spherical probe representing the solvent. The calculation was performed using the “gmx sasa” tool from the GROMACS package, which analyzes this property throughout the MD’s trajectory. Thus, the average value obtained for Model-1 was an area of approximately 233 nm^2^, for Model-2 we obtained ∼ 469 nm^2^, for Model-3 we obtained ∼ 707 nm^2^, for Model-4 we obtained ∼ 934 nm^2^, and finally, for Model-5, we obtained a solvent-accessible area of ∼ 1164 nm^2^. As we can observe, the values found for Models 2 to 5 are very close to multiples of the value obtained for Model-1, indicating that the structures maintain a standardized surface structure.

### 3.2 Coulomb and Lennard-Jones Interaction Energy

Coulomb energy and Lennard-Jones energy are complementary and fundamental factors for evaluating the cohesion of peptide nanostructures, especially in assessing their interactions with the aqueous environment, which defines their potential applications. Playing crucial roles in intermolecular interactions, these energies allow us to evaluate the stability and conformation of these structures during MD simulations. Coulomb energy measures the electrostatic interaction between charged particles and is essential for evaluating how the net negative charge of peptides affects fiber cohesion, maintaining the shape and integrity of structures, as in protein formation, where opposite charges between amino acid groups can stabilize the three-dimensional configuration. This information is highly valuable for assessing applications in nanomedicine and materials science. On the other hand, the interaction energy described by the Lennard-Jones term accounts for interactions between particles regardless of their charge, considering both attractive and repulsive forces. This potential is used to model van der Waals forces, which are fundamental in the formation and stability of nanostructures. These interactions are responsible for the attraction between nearby molecules and can influence the aggregation of peptides in the self-assembly of fibers. Furthermore, the interactions modeled by the Lennard-Jones potential affect the dynamics of molecules in solutions (especially in water) or on surfaces, influencing how nanostructures move, interact, and organize within complex systems.

Figure 5 shows the behavior of Coulomb and Lennard-Jones energy for the five studied models, comparing the interactions between the peptides, between the peptides and potassium ions, and between the peptides and water molecules. For model-01, the Coulomb energy between the peptides is approximately −7 × 10^3^ kJ/mol, a value that remains consistent across all other structures (variations of less than 1%), highlighting that the smaller structure can adequately represent the energetic aspect of the larger structure. The results show that although we have a structure with charged peptides aggregated very closely, the Coulomb interaction energy is quite attractive and may indicate high structural stability of the fiber, even when compared to other structures. Previous studies on nanofibers formed by the peptides K_2_(SL)_6_K_2_,^17^ G_3_A_3_V_3_I_3_K_3_,^2^ and K_3_I_3_V_3_A_3_G_3_ ^2^ showed that the Coulomb energy was approximately −4.3 × 10^3^ kJ/mol. Therefore, we observe that the values found for the Coulomb energy between the peptides are approximately 1.63 times greater than those found in previous works with the same nanostructure but with different peptides. This high electric interaction energy may be related to the efficient way in which the peptides aggregate, forming a dimer with a hydrophilic outer layer and a hydrophobic inner layer, combined with the stacking of β-sheets that favor structural characteristics governed by hydrogen bonding interactions. It is also important to highlight that the energetic interaction between tryptophan residues (the hydrophobic region of the peptide) accounts for approximately 38% of the peptide–peptide interaction in the nanofibers and is related to the proximity of the C–N ring of the tryptophan residue, which is stacked inside the fiber (see Figure 1c and 1d).

**Figure 5:**
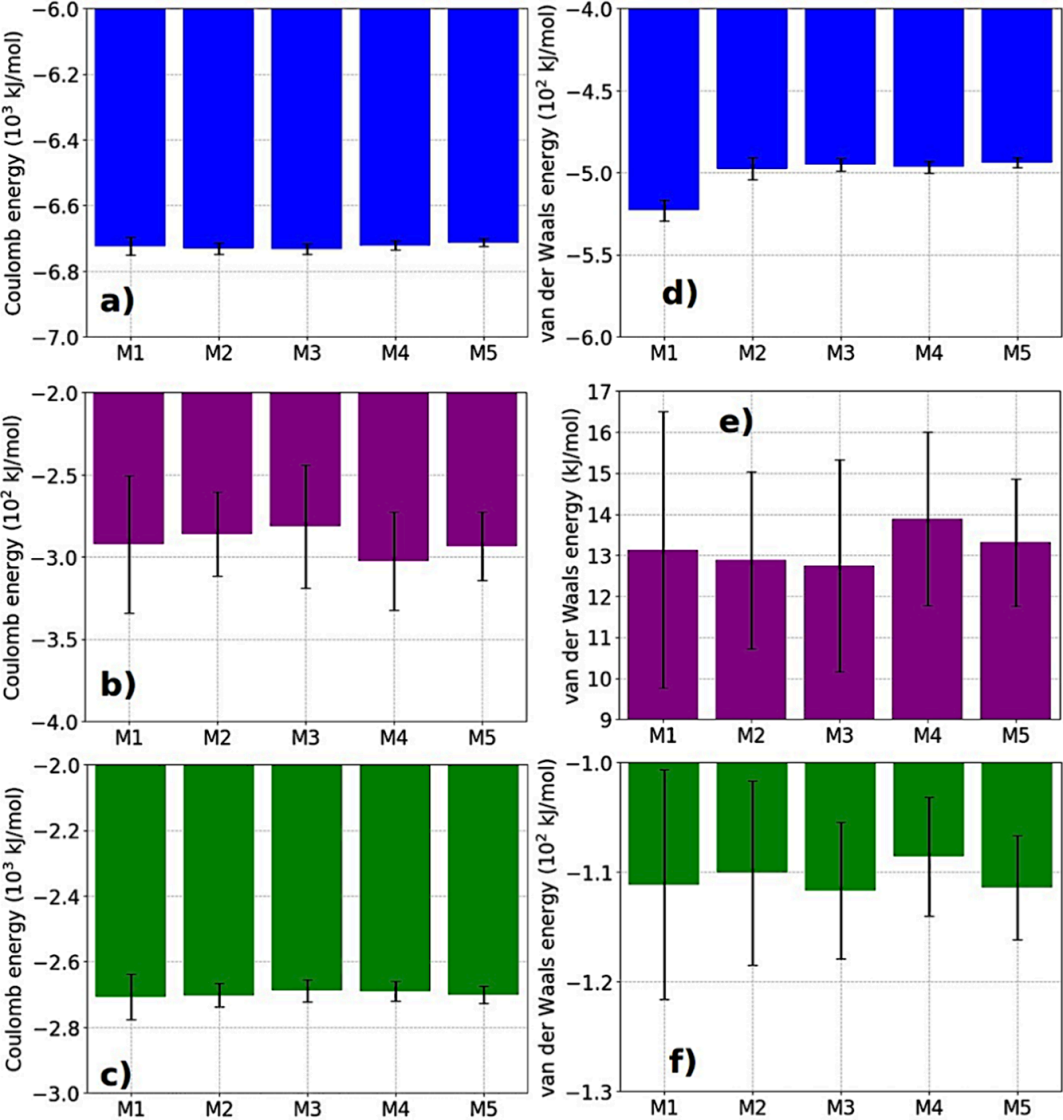
Coulomb interaction energy for (a) peptide–peptide (in blue), (b) peptide–ions (in purple), and (c) peptide-water molecules (in green), per peptides, for all Model-XXs. Lennard-Jones interaction energy for (d) peptide–peptide (in blue), (e) peptide–ions (in purple), and (f) peptide-water molecules (in green), per peptides, for all Model-XXs.

For the Coulomb interaction energy between the peptides and potassium ions, energies ranging from −300 to −380 kJ/mol per peptide were obtained (with variations of up to ∼ 4% between the models compared to model-01). This result indicates that the peptide structure has a high energetic potential for ionic capture of opposite charges, which could indicate a potential application for the efficient transport of charged drugs. The analysis of electrostatic interactions between each residue and the K^+^ ions reveals that glutamate plays a fundamental role in the adsorption of ions onto the nanofiber. With an average value close to −310.88 kJ/mol, this interaction is extremely strong, indicating that glutamate residue, being a negatively charged residue, significantly attracts K^+^ ions. Serine residue also contributes to this interaction, but with a much lower intensity (around −12.45 kJ/mol), suggesting a secondary role in the adsorption process. Tryptophan residue presents the weakest electrostatic interaction (around −5.81 kJ/mol), indicating that its influence on the electrostatic interaction with K^+^ ions is minimal. But, it is important to highlight that the high mobility of the ions is related to the variation in the peptide–ion interaction values, as observed in Figure 5.

Finally, the Coulomb interaction energy between the peptides and water molecules indicates values close to −2700 kJ/mol per peptide (with variations of up to 0.70% between the models). This high Coulomb interaction between the peptides and water can be attributed to the serine residues that remained in the outer region of the strands, favoring an interaction that indicates a large number of HBs on the surface of the structure. Compared to the nanofibers from previous studies formed by the peptides K_2_(SL)_6_K_2_,^17^ G_3_A_3_V_3_I_3_K_3_,^2^ and K_3_I_3_V_3_A_3_G_3_,^2^ our results showed an increase of 1.71 times compared to the nanofiber formed by K_2_(SL)_6_K_2_, 2.50 times compared to the nanofiber formed by G_3_A_3_V_3_I_3_K_3_, and 2.64 times greater compared to the structure formed by K_3_I_3_V_3_A_3_G_3_.

For the Lennard-Jones (LJ) interaction energy between the peptides, we found values ranging from approximately −500 kJ/mol for Model-01 to −490 kJ/mol for Model-05, again showing that the size of the simulated configuration has no impact on the energetic aspect of this structure. We can observe that the LJ interaction between peptides has a high value (about 1/14 of the Coulomb interaction energy), which contributes to the formation of more stable structures with closer side chains. Part of this LJ interaction between the peptides is due to interactions among the tryptophan (W) residues and their side chains, which possess a ring that favors π−π interactions. This morphology was maintained throughout the simulation and induces a high LJ interaction between the peptides, especially in the hydrophobic region.

The Lennard-Jones interaction energy between the peptides and potassium ions was found to be positive, indicating a repulsive interaction, with average values close to 13 kJ/mol per peptide. These values do not significantly impact the peptide–ion attraction due to the high Coulomb interaction energy between them, which is about 20 times greater. Nonetheless, these positive values may be related to the proximity of the ions to the peptides, as the repulsive term in the Lennard-Jones interaction becomes more significant. For LJ interaction energy between residues and K^+^ ions we found: (a) glutamate residue has an average value of approximately 14.77 kJ/mol, suggesting a slight repulsion compared to the strong electrostatic attraction observed earlier; (b) for the serine residue, the observed E_LJ_ value is around 0.68 kJ/mol, indicating a weak vdW interaction with the K^+^ ions; (c) on the other hand, tryptophan residues present a negative value for this interaction (−0.22 kJ/mol), indicating a slight attraction between this residue and K^+^ ions. However, since these values are much smaller than those observed for Coulomb interactions, we can conclude that K^+^ ion adsorption is primarily governed by electrostatic forces.

For the Lennard-Jones interaction energy between the peptides and water molecules, the average values found are around −110 kJ/mol per peptide (with variations of ∼ 2% between the models). As expected, when peptides are in an aqueous environment, the nonpolar parts tend to cluster together to minimize their interaction with water; thus, the aggregated peptide structure exhibited a considerably low LJ interaction energy compared to the peptide-water electric interaction, representing only about 1/25 of the Coulomb interaction. Compared to the nanofibers from previous studies formed by the peptides K_2_(SL)_6_K_2_,^17^ G_3_A_3_V_3_I_3_K_3_,^2^ and K_3_I_3_V_3_A_3_G_3_,^2^ our results for the LJ interaction between the peptides and water show variations of 0.5 times for the nanofibers formed by K_2_(SL)_6_K_2_, 0.49 times for the nanofibers formed by G_3_A_3_V_3_I_3_K_3_, and 0.43 times for the nanofibers formed by K_3_I_3_V_3_A_3_G_3_. This indicates variations of up to 57% compared to these works. The supporting material provides details of the values presented in Figure 5.

### 3.3 Dynamic, Energetic and Structural Analysis of Hydrogen Bonds

In peptide nanostructures, HBs play a crucial role in their formation. Previous studies have shown that these structures exhibit a specific orientation of HBs, characterizing the self-assembled structure. They are highly cooperative and cumulative, making them essential for maintaining the three-dimensional conformation of peptide molecules and nanostructures. The duration of HBs is also a crucial factor in theoretically inferring how much they are responsible for maintaining the self-assembled structural cohesion and will be analyzed in this context. The longer this specific interaction lasts, the more stable the structure maintained by these bonds. In MD simulations, monitoring the hydrogen bond lifetime can also reveal regions of greater or lesser structural flexibility, indicating how the structure might behave. We emphasize that the time step at which this information is obtained is related to the time step used for integrating the system’s equations of motion (0.001 ps) and the frequency with which the configurations are saved for statistical analysis (every 2 ps). Thus, the presented values are computed based on this temporal information, and the results correspond (in ps or ns) to the lifetimes of the HBs obtained from configurations spaced at this simulation time interval. Figure 6 shows the orientation of HBs along the peptide strand of Model-05, where we can observe that the bonds occur along the ribbon-like structure, in the *z*-axis, contributing to the maintenance of the stacked sheet (dimers) structure. Figure 6 also shows the cavity of the peptide structure embedded within the aqueous medium. It is notable that the structure isolates the water molecules in the region enclosed by its hydrophilic surface, maintaining a high degree of internal organization characterized by strong interactions between hydrophobic amino acids. In highlight (Figure 6c), we present the HBs shown in blue, aligned along the backbone of the nanoribbon, maintaining the peptides’ stable orientations perpendicular to its length.

**Figure 6:**
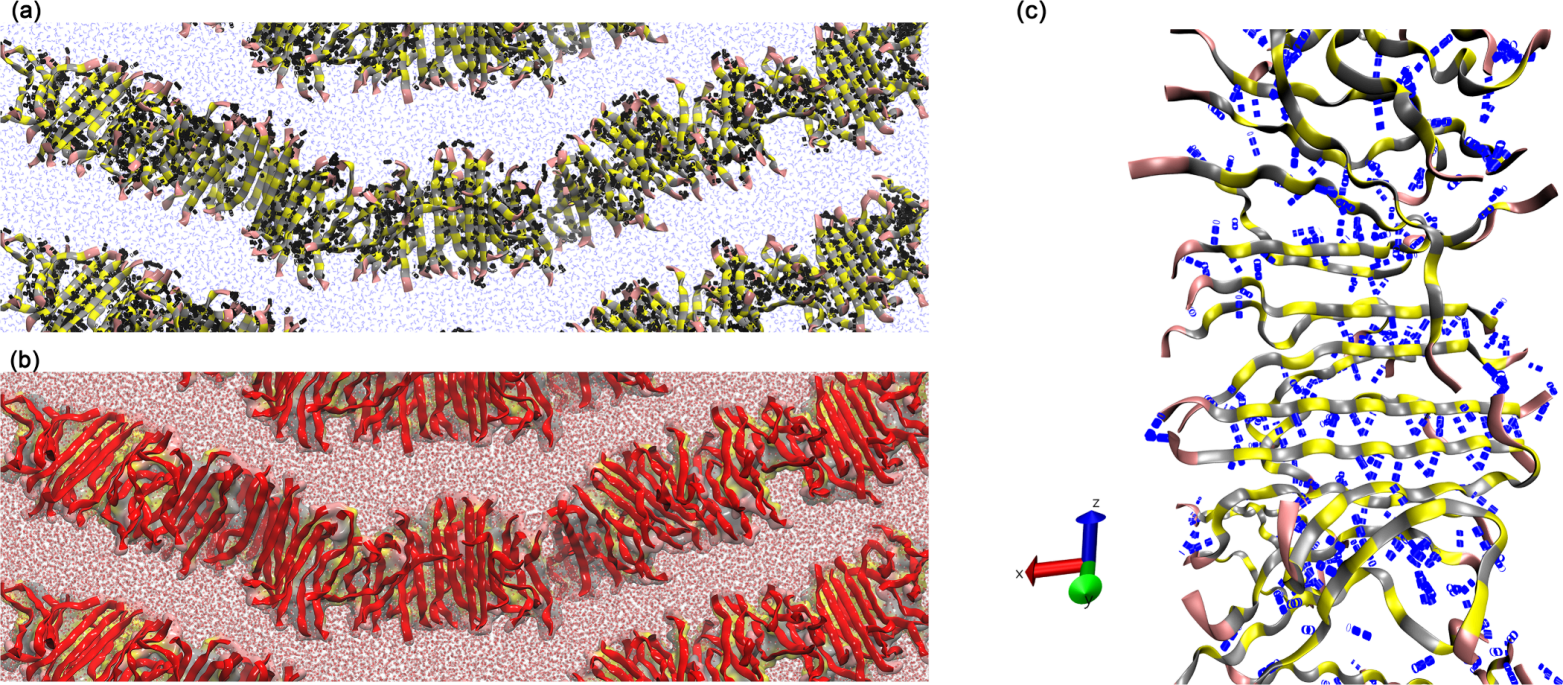
(a) Representation of the hydrogen bonds along the simulated peptide chain for Model-05. The peptides are represented as ribbons, with pink representing glutamate residues, yellow representing serine residues, gray representing tryptophan amino acids, black traces representing hydrogen bonds, and blue representing water molecules. The hydrogen bonds between peptides along the nanofiber hold the peptides together, maintaining the cohesiveness of the nanofiber; (b) Representation of the cavity formed within the solvent environment, characterizing the region that makes up the nanofiber. The water molecules are shown in red, and the peptide is in red ribbons. The colored surface containing the ribbons represents the cavity of the nanofiber in solution; and (c) Demonstration of the preferential alignment of HBs along the nanoribbon (blue tubes). As can be observed, the HBs have an orientation aligned with the backbone of the nanostructure, while the peptides are perpendicular to its length.

Table 02 shows the average number ( N ), average lifetime ( T , in ps), and hydrogen bond dissociation energy (Δ G , in kJ/mol) obtained for a radius of up to 0.35 nm and an angle of up to 30°. For interactions between peptides, our results indicate that N is approximately 7 HBs per peptide, with slight variations between models, suggesting that regardless of the size of the simulated structure, the average number of HBs is a practically characteristic factor of the structure that maintains its idealized formation. The interactions between peptides can be observed in even greater detail by analyzing the number of HBs between residues. In this case, the results show that the average number of HBs per peptide for glutamate-glutamate residues is around 1.5–1.6 HBs; for glutamate-tryptophan residues, we observe a maximum of 0.5 HBs per peptide; for GLU-SER, the values range between 0.7 and 0.8 HBs per peptide, depending on the studied Model-XX. For serine–serine residue interactions, the average values indicate approximately 1 HB per peptide, while tryptophan- tryptophan shows a lower average of around 0.4–0.5 HBs per peptide. The interaction between serine-tryptophan stands out as the most prevalent in the nanoribbon, with an average number of HBs per peptide ranging between 2 and 3. This interaction plays a key role in maintaining the packed structure, ensuring that each dimer (Figure 1c) remains laterally stacked along the nanoribbon’s length.

**Table 02:** Average Number of HBs per Peptide Unit ( N ), Lifetime ( T , in ps), and Breaking Energy of Hydrogen Bonds (Δ G , in kJ/mol) Obtained from Van Der Spoel and Luzar-Chandler Theory^44–46^

	Model-01	Model-02	Model-03	Model-04	Model-05
# HB _Peptide–Peptide_	7.2 ± 0.04	6.9 ± 0.2	7.4 ± 0.1	6.7 ± 0.4	6.7 ± 0.4
# HB _Peptide‑Water_	64.2 ± 0.1	63.6 ± 0.5	63.5 ± 0.2	63.5 ± 0.8	63.7 ± 0.7
T _Peptide–Peptide_	129.6	322.7	869.7	1680.9	2030.0
T _Peptide‑Water_	3.7	10.1	54.1	41.7	40.8
Δ G _Peptide–Peptide_	16.6	18.8	21.3	22.9	23.4
Δ G _Peptide‑Water_	7.7	9.5	14.4	13.8	13.7

For interactions between peptides and water, the average values of N are around 63 HBs per peptide (the variations between models did not exceed 1.5%), indicating that the size of the simulated structures also has little influence on the interaction between the peptide and water, considering only the average number of HBs. We emphasize that this value obtained for N between peptide-water is considerably high per peptide unit, highlighting the hydrophilicity of the structure. The analysis of HBs between the peptides and water molecules can be observed for each of the residues that make up the peptide (E_2_(SW)_6_E_2_) in the nanofiber. Of the total 16 residues, only 6 face the interior of the nanofiber, as shown in Figure 1c. However, this positioning does not inhibit interaction with water molecules; on the contrary, our results show that the nanofiber undergoes hydration. The remaining amino acids are positioned on the surface of the nanofiber, with 4 of them containing charged regions that favor a higher number of hydrogen bond interactions. Additionally, the -NH_2_ and −COOH termini characteristic of the peptide also promote HBs between the water molecules. The results indicate that glutamate residues form about 9 HBs each, a number that aligns with their net charge, which favors greater electrical interaction, as well as their structural position at the ends of the nanofiber, exposing the amino acid more and increasing its direct interaction with the surrounding solvation layer. Serine residues form about 3 HBs each, while tryptophan residues form about 1.5 HBs each.

Another important analysis to be conducted is the duration of the HBs. Our results for the interactions between the peptides show an influence of the size of the analyzed model, indicating that smaller models tend to have a similar average number statistic as larger models, but fail in comparisons involving the duration of these interactions. For Model-01, we obtained a T close to 130 ps; for Model-02, this value is about 322 ps; for Model-03, we obtained about 870 ps; for Model-04, we have interactions that remain uninterrupted for about 1.7 ns; while for Model-05, this interaction can last up to 2.0 ns (Figure S3 shown autocorrelation curves for hydrogen bond statistic between peptide–peptide for different models). From these results, we observe that the HBs have a duration on the order of nanoseconds as the length of the chains increases, showing a variation of over 1000% when comparing the largest and smallest models studied. Such results indicate that simulations with longer structures of these peptides exhibit a more flexible, dynamic, and consequently more stable structure than the others. This may be related to the fact that the peptide structure requires a specific length for its natural dynamics, and in smaller structures simulated with periodic boundary conditions (PBCs), this dynamic is compromised due to the small length of the structure along the *z*-axis, especially since one end of the structure is tethered to the other end due to PBCs. However, it is important to highlight that the thermal effects on the packing of self-assembling peptides have shown a significant reduction/increase in the lifetime of HBs when the temperature is increased/reduced, as observed in previous studies.^17^ For the duration of HBs between peptides and water, we also observed a slight dependence on the size of the peptide fiber structure. Our results showed that the values of T for peptide-water range from 10 ps (Model-01) to about 41 ps (Model-05). For the hydrogen bond breaking energy (Δ G ) between the peptides, our results show a trend toward convergence of the Δ G value as the peptide strand grows, with results ranging from approximately 17, 19, 21, 23, to 23 kJ/mol, respectively for Models-01 to 05. From these results, we can observe that larger structures (when analyzed from the perspective of hydrogen bond dynamics of the peptide–peptide) may appear more stable than smaller structures. On the other hand, the results obtained for the peptide-water interactions (respectively 7.7, 9.5, 14.4, 13.8, and 13.7 kJ/mol for each Model-XX) indicate that the system’s length should also be considered during the computational simulation to correctly identify factors in the peptide-water interaction that may highlight the hydrophilic nature of the system. It is also worth noting that the existing β-sheets favor the existence of lateral interactions between peptides through HBs. Evidence of this characteristic is shown by the high number of HBs in the system, which averages about 7 HBs per peptide. On the other hand, another packing is evident in the interaction between tryptophan (W) residues, which are favored by interactions in the stacking of their C–N rings. While the first characteristic can be related to the maintenance of the nanofiber’s length, the second can be associated with the behavior of the hydrophobic region of the nanofiber, which tends to shield itself from the aqueous medium.

### 3.4 Ramachandran Plots

The Ramachandran plot maps the φ vs ψ (phi vs psi) dihedral angles of a polypeptide chain, revealing the possible conformations of the peptide bonds. It helps visualize the preferential conformations and the most stable or flexible regions of the molecule during a simulation. Specific regions of the plot are associated with secondary structures, such as α-helices and β-sheets. α-helices typically appear in areas with – 60° < φ < – 50° and – 45° < ψ < – 30°, while β-sheets are found in regions with – 150° < φ < – 130° and 130° < ψ < 150°. These areas indicate the ideal conformations for these structures, aiding in the structural analysis of proteins and peptides. In Figure 7, we present the Ramachandran plot, which shows the density of the most favorable regions for the φ vs ψ angles, measured together, obtained from configurations extracted from the MD simulations. Figure S2 (in the Supporting Information) complements these plots, showing the Ramachandran plot in 3D, highlighting the intensity of points in the preferential φ vs ψ regions shown in Figure 7. We can observe that, for Model-01 (Figure 7a), the glutamic acid (E-residue), located at the ends of the E_2_(SW)_6_E_2_ peptide, has a lower maximum count for the φ vs ψ angles compared to Models-03 and 05 (Figures 7b and 7c). This may indicate that this region is less flexible in Model-01, which is a structural characteristic of the simulated nanostructure with this specific length along the *z*-axis. For Model-01 (Figure 7a), the serine (S-residue) and tryptophan (W-residue) show only a small region with φ vs ψ angles recorded in the diagram, suggesting that these amino acids are more rigid during the simulation, predominantly adopting a β-sheet structure with little flexibility. Thus, the central region of the peptides (S and W residue) remains fixed, without folding during the simulation, characterizing torsions that lead the nanofiber to intertwine. Figure S2 highlights a few occurrences of φ vs ψ values in other regions of the plot, but these counts are considerably lower than the φ vs ψ counts that characterize the predominantly β-sheet structure.

**Figure 7:**
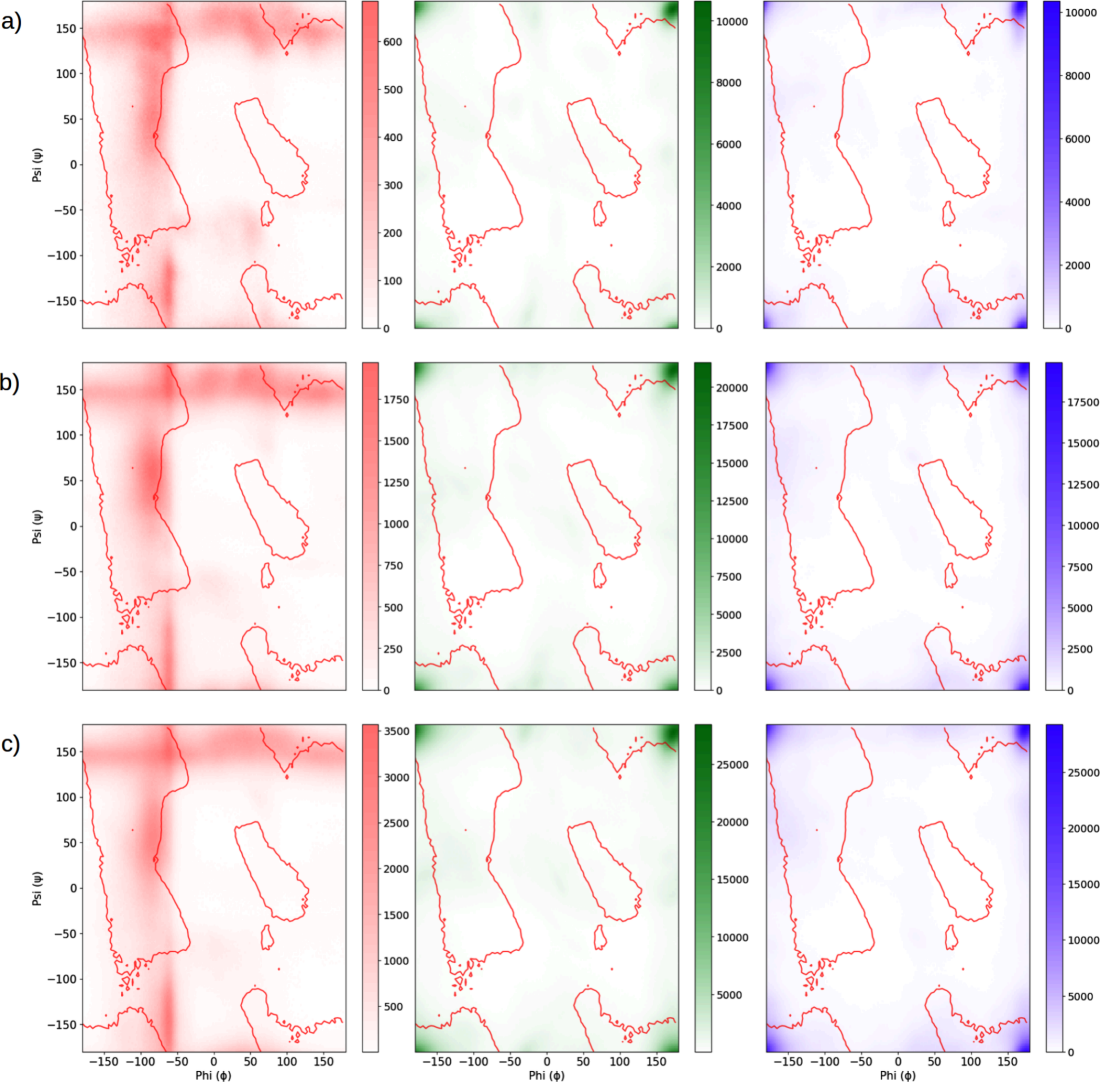
Representation of the Ramachandran plots for models (a) Model-01, (b) Model-03, and (c) Model-05. In red for E residues, in green for S residues, and in purple for W residues. The regions (Psi, Phi) with higher population are shown with more intense coloring. The curves drawn in the plots represent the (Psi, Phi) regions that characterize the peptide structure as β-sheet or α-helix for the studied amino acids. In the supporting material (Figure S4), we present these plots in 3D, highlighting the most populated regions as peaks on the *Z*-axis of the Ramachandran plots.

For Model-02, we observe that the E-residue has a higher maximum count compared to Model-01, suggesting that this end region of the peptide in Model-02 became less flexible with the sheet’s growth, resulting in more φ vs ψ records in a specific region. However, the structure is still predominantly of the β-sheet type. For the serine residues (S), we also see an increase in this maximum count, showing that this region became more fixed in the β-sheet structure. For the tryptophan residue (W-residue), the count was slightly lower than in Model-01, possibly indicating a bit more flexibility. This slight flexibility may be associated with the wavy shape of the fiber, a characteristic observed only in models with longer simulated longitudinal lengths. Model-05 (Figure 7c) follows a structural trend similar to Model-02, with the E-residue standing out for its lower φ vs ψ angular flexibility and maintaining its structure predominantly as a β-sheet, especially in the serine (S-residue) and tryptophan (W-residue). Overall, the Ramachandran plots confirm the sheet-like structure of two aggregated and stacked peptides in a β-sheet form along the fiber growth axis, with a rigid angular region, – (SW)_6_–, and slight freedom at the ends, E_2_– and – E_2_ terminations. These characteristics, combined with the previous findings, make the self-assembled peptide nanostructure an excellent rigid structure, cohesive structure—hydrophilic on its surface and hydrophobic in its inner region. Its high surface charge makes it highly effective as a drug or ion carrier/collector, with great potential for applicability.

### 3.5 Mean Square Displacement (MSD)

The Mean Square Displacement (MSD) is a dynamic property that quantifies the dispersion of a particle in a system. This quantification is related to the average of the squared displacements relative to a previous position during the MD simulation and highlights the mobility of the peptide structures that make up the system, providing insights into the behavior of the peptides throughout a simulation. The calculation is performed using Einstein’s equation, which defines the diffusion coefficient ( D _A_) for particles of type A. For Model-01, the $D_{01}$ found was (0.9 ± 0.4) × 10^–7^ cm^2^/s, for Model-02 this value was close to $D_{02}$ = (0.8 ± 0.2) × 10^–7^ cm^2^/s, for Model-03 we found values nearly twice as high, $D_{03}$ = (1.5 ± 0.5) × 10^–7^ cm^2^/s, while Model-04 showed values similar to Model-03, $D_{04}$ = (1.2 ± 0.9) × 10^–7^ cm^2^/s. Finally, for Model-05, we found higher results than the other models, $D_{05}$ = (2.0 ± 0.3) × 10^–7^ cm^2^/s, indicating that the increase in peptide fiber length introduces greater system mobility, as previously noted (Figure S5 shown the mean-squared displacement curves for all models). It is important to emphasize that this increase in molecular mobility could lead to fiber rupture or fragmentation; however, the larger models also demonstrate a long hydrogen bond lifetime, allowing the peptide chains to maintain their characteristic structure within the fiber, dynamically preserving the nanostructure.

## 4 Conclusion

Based on the presented results, we observe that the variations in Coulomb energy between the peptides in the studied strands are less than 1%, indicating that all models exhibit similar interactions. Additionally, the Coulomb interactions between the peptides and the ions showed variations of up to 3.7%, while the interactions between the peptides and water had variations of less than 1% when comparing the five models studied. The Lennard-Jones energy between the peptides showed a variation of approximately 7% among the models, with the shorter one yielding the highest result. The Lennard-Jones interactions between the peptides and the ions are noteworthy for being repulsive, which may be related to the excessive proximity between these particles but does not compromise the attractive electric interaction. The energetic description of these structures formed by the E_2_(SW)_6_E_2_ peptide suggests strong cohesion between peptides, between peptides and ions, and also between peptides and water, being considerably greater than that reported for other peptide structures in the literature.

The energy values indicate significant differences between the K_2_(SL)_6_K_2_ and E_2_(SW)_6_E_2_ systems, particularly in the electrostatic and van der Waals interactions. The E_2_(SW)_6_E_2_ system exhibits significantly more negative Coulomb energy values for peptide–peptide interactions, around −6724 kJ/mol, while the K_2_(SL)_6_K_2_ system shows values close to −4318 kJ/mol. This indicates much stronger electrostatic interactions between the peptides in the E_2_(SW)_6_E_2_ model. Furthermore, the Lennard-Jones energy for these interactions is also more negative in E_2_(SW)_6_E_2_, with average values around −500 kJ/mol, compared to −340 kJ/mol for K_2_(SL)_6_K_2_, suggesting more intense van der Waals interactions and a more stable packing of the peptides. Regarding peptide–ion interactions, the structure composed of E_2_(SW)_6_E_2_ shows a more negative Coulomb energy, approximately −290 kJ/mol, while the K_2_(SL)_6_K_2_ structure has values close to 1.5 kJ/mol, practically insignificant compared to the value found for the nanostructure studied in this work. This indicates that E_2_(SW)_6_E_2_ interacts more strongly with the ions present in the solution, which may influence its structural organization and stability, especially due to ionic strength. For Lennard-Jones interactions, the values are small in both systems, but the structure composed of E_2_(SW)_6_E_2_ shows slight repulsion. In peptide-solvent interactions, the E_2_(SW)_6_E_2_ structure again exhibits more intense Coulomb energies, close to −2700 kJ/mol, indicating more favorable interactions with water molecules, compared to average values of about −1580 kJ/mol for structures formed by K_2_(SL)_6_K_2_. Overall, the energy data suggest that E_2_(SW)_6_E_2_ peptides form more cohesive and stable structures due to stronger interactions, and also exhibit greater interaction with ions and solvent. This may suggest that this system is more suited for the formation of strongly interconnected nanofibers, while systems composed of K_2_(SL)_6_K_2_ may present a more fragile structure, less influenced by electrostatic interactions with the environment.

Based on the mass density profile projected along the fiber length (*z*-axis of the simulation), it can be concluded that the mass of the nanotapes is distributed throughout the nanostructure, with an average density of ∼ 300 kg/m^3^ in some regions, indicating good structural consistency along the entire simulated fiber, characterizing structural integrity. However, regions of mass density decrease along the length of the nanofiber can be observed. This decrease in mass density can exceed 50%, but no average mass density of zero was observed along this axis, which would characterize a local rupture of the nanofiber during a long computational simulation time. This high cohesion can be justified by the strong interaction energy and also by the high number of peptide–peptide HBs. In this case, the studied models show variations of ∼ 7% in the average number of HBs between the peptides that make up the fiber and ∼ 1% between the peptides and water molecules, indicating that the size of the tapes has little influence on this analysis. However, despite the average number of HBs being practically stable among the models, we noted that as the length of the tapes increases, the HBs between the peptides become more durable, reaching up to 2 ns (for larger models) and characterized by a breaking energy barrier of ∼ 23 kJ/mol. Together, the results obtained from the Ramachandran plots show that the central regions of the peptide are more rigid compared to the terminal regions, predominantly adopting β-sheet configurations, which are essential for maintaining the laminar/fiber characteristics of the system. Finally, through the analysis of the MSD, we conclude that as the peptide strands increase in length, there is a significant rise in mobility, with the longest model exhibiting the highest MSD, approximately two times greater than that of the shortest model. This increased mobility suggests that the strands become more flexible with their size; however, they do not lose their characteristic structure, especially due to the high number of HBs, extended lifetimes, and elevated interaction energy between peptide–peptide interactions. Although we have exclusively used K^+^ for neutralization, the choice of cation can significantly influence the organization and stability of the nanofibrils. Monovalent cations with distinct ionic radii (e.g., Na^+^ or Li^+^) can penetrate narrower cavities, altering the packing and hydrogen bond dynamics, while multivalent cations (Ca^2+^, Mg^2+^) tend to form salt bridges between peptide chains, reinforcing interchain cohesion at the cost of reducing ionic dynamics. In biomedical contexts, these effects are critical: Ca^2+^ and Mg^2+^, abundant in certain biological fluids, can modulate peptide conformations and drug release behavior. Therefore, future studies should systematically evaluate different ionic species to optimize the mechanical properties, biocompatibility, and performance of these nanomaterials in therapeutic molecule transport or controlled release applications.

## Supplementary Material



## Notes

Complementary details on molecular dynamics methodologies, including unsuccessful nanofiber modeling strategies; potential energy profiles for all peptide nanofiber models (Figure S1); temperature stability analysis across simulations (Figure S2); summary table of average peptide–peptide, peptide–ion, and peptide–water interaction energies (Table S1); hydrogen bond lifetime autocorrelation functions for different models (Figure S3); 3D Ramachandran plots illustrating secondary structure preferences (Figure S4); mean-squared displacement curves of peptides highlighting diffusion behavior in each model (Figure S5) (PDF)
