# Supplementary material for: Molecular Dynamics Simulations of Self-Assembled E2(SW)6E2 Peptide Nanofibers: Implications for Drug Delivery and Biomimetic Material Design
Source: ACS Phys Chem Au. 2025 May 8;5(3):302–15. doi: 10.1021/acsphyschemau.5c00028 (PMC12123548; doi:10.1021/acsphyschemau.5c00028)
Supplement: Supplementary file 1 [file pg5c00028_si_001.pdf]

SUPPORTING INFORMATION

Molecular Dynamics Simulations of Self-Assembled $E_2(SW)_6E_2$ Peptide Nanofibers: Implications for Drug Delivery and Biomimetic Material Design

*Karinna Mendanha[a] and Guilherme Colherinhas[a]**

[a] Instituto de Física. Universidade Federal de Goiás. 74690-900. Goiânia. GO. Brazil.

** Corresponding author. E-mail address: gcolherinhas@ufg.br*

COMPLEMENTARY METHODOLOGY:

Unsuccessful strategies during nanofiber modeling: Some of our initial attempts to model the nanofiber were unsuccessful during the early MD simulations. In these tests, we tried to model the system by first solvating the system completely and varying MD simulations in NPT and NVT ensembles. In these tests, we observed that the system lost its characteristic nanofiber/nanostructure, with the peptides separating and scattering without organization within the box, resulting in only amorphous aggregates. We assessed that the ions did not aggregate to the structure before it fragmented, indicating that the ionic strength did not play a significant role in maintaining the structure's cohesion in this first strategy.

We also attempted to model the nanofibers by conducting the initial MD simulations with the system fully solvated in water and only using the NVT or NPT ensemble. In both tests, the results were unsuccessful due to issues such as lack of freedom in the NVT process and difficulty in adjusting the simulation box in NPT simulations when the nanofibers were not yet cohesive, resulting in structures that once again formed amorphous aggregates without apparent periodicity.

In all these cases, the structure quickly disintegrated after only a few simulation steps, demonstrating a very aggressive effect of fiber hydration on its structural integrity. We therefore considered the possibility of initially adding ions to the nanostructure and then fully solvating the system. This strategy involves the initial action of ionic strength to promote cohesion in the nanostructure and is described in the manuscript text as the successful approach we used to develop the proposed work.

Additionally, structural initial configurations distinct from those represented in Figures 1c and 1d were also simulated. These configurations were subjected to the same initial simulation conditions described here and were unsuccessful. The successful initial configuration (presented in the manuscript) is the one with the best alignment of interactions between the peptides, reducing empty spaces and maximizing the structural fit that favors hydrogen bonding along the nanofiber.

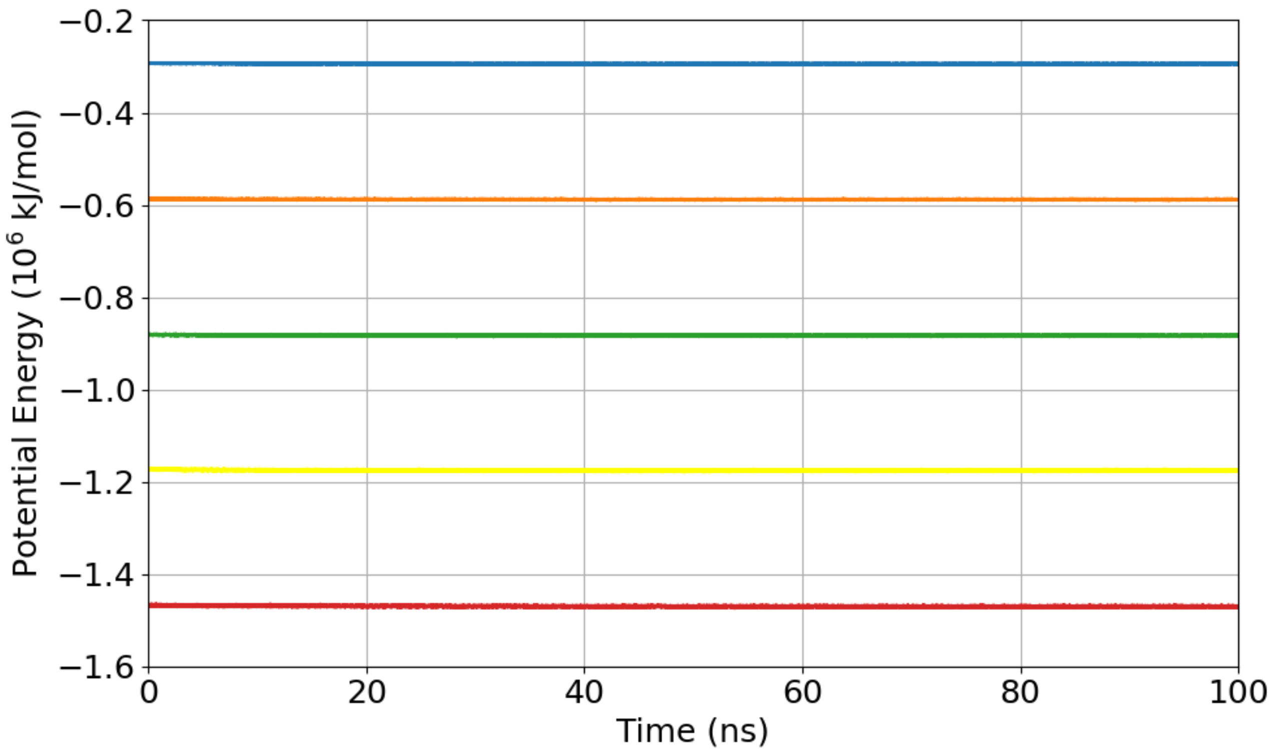

Figure S1. Potential energy profiles of all systems (Models 01–05) during the production stage. The figure presents the five datasets together, allowing a comparative view of the stability and fluctuation patterns among the different models. The colors represent the different models as follows: blue corresponds to Model-01, orange to Model-02, green to Model-03, yellow to Model-04, and purple to Model-05.

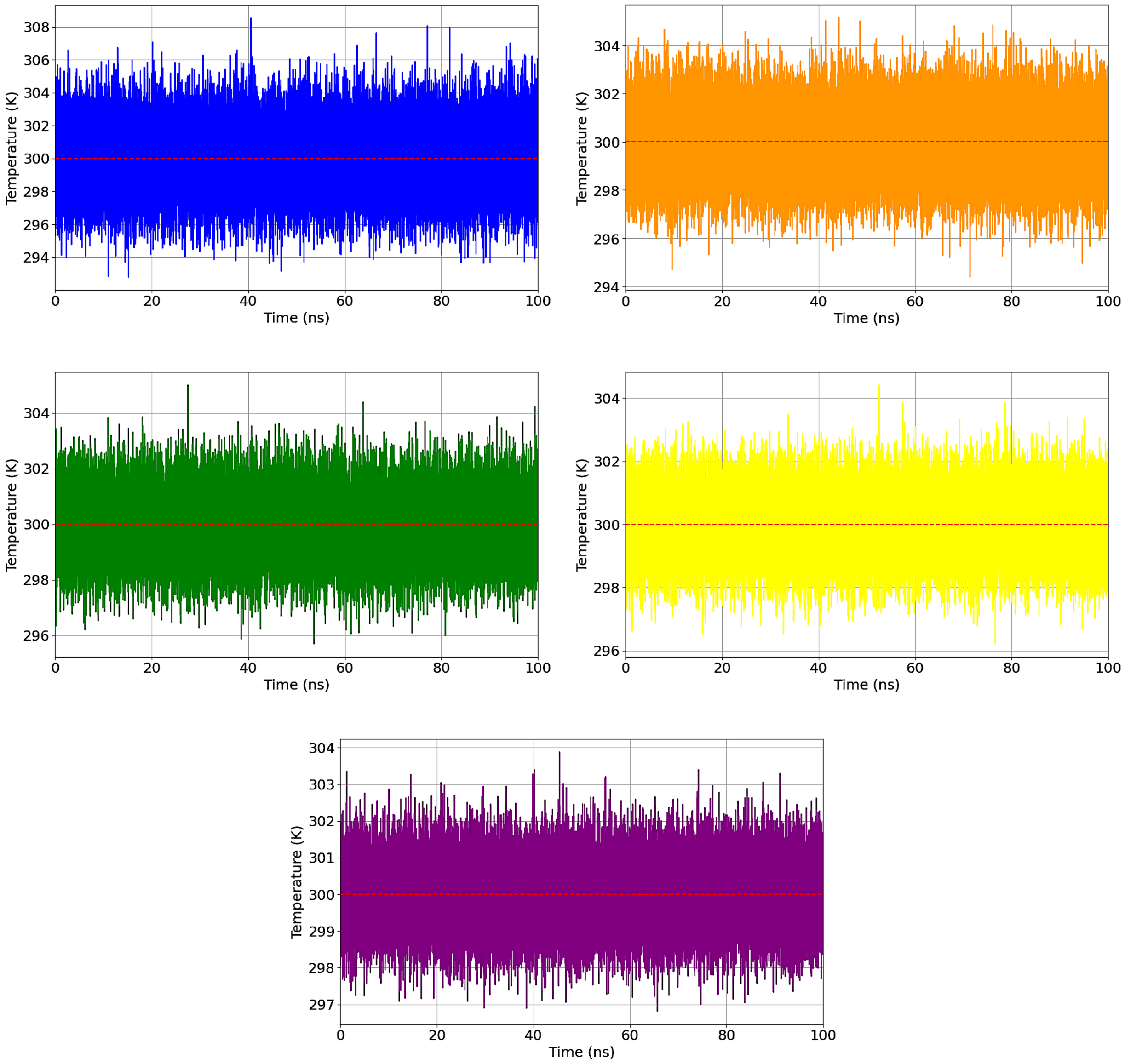

Figure S2. Temperature profiles of all systems during the production stage. Each plot corresponds to one dataset, representing Models 01 to 05 individually, and highlights the temperature stability throughout the simulations. The colors represent the different models as follows: blue corresponds to Model-01, orange to Model-02, green to Model-03, yellow to Model-04, and purple to Model-05.

Table S1: Average Coulomb and Lennard-Jones energy interaction (in kJ/mol per peptide).

Model-01	***Coulomb***		***Lennard-Jones***	
	E_C	***RMSD***	***E_{LJ}***	***RMSD***
Pep-Pep	-6724.05	26.76	-522.96	6.21
Pep-Íon	-292.30	41.82	13.13	3.37
Pep-Water	-2707.32	68.72	-111.14	10.49
Model-02	***Coulomb***		***Lennard-Jones***	
	E_C	***RMSD***	***E_{LJ}***	***RMSD***
Pep-Pep	-6731.50	17.15	-497.55	6.71
Pep-Íon	-286.03	25.62	12.88	2.15
Pep-Water	-2701.80	35.81	-110.08	8.40
Model-03	***Coulomb***		***Lennard-Jones***	
	E_C	***RMSD***	***E_{LJ}***	***RMSD***
Pep-Pep	-6732.68	16.19	-495.10	3.97
Pep-Íon	-281.48	37.53	12.75	2.58
Pep-Water	-2688.48	32.87	-111.66	6.24
Model-04	***Coulomb***		***Lennard-Jones***	
	E_C	***RMSD***	***E_{LJ}***	***RMSD***
Pep-Pep	-6721.50	14.77	-496.51	3.70
Pep-Íon	-302.52	29.89	13.89	2.11
Pep-Water	-2689.20	30.50	-108.58	5.44
Model-05	***Coulomb***		***Lennard-Jones***	
	E_C	***RMSD***	***E_{LJ}***	***RMSD***
Pep-Pep	-6712.63	12.08	-493.84	3.08
Pep-Íon	-293.50	20.81	13.31	1.55
Pep-Water	-2701.23	26.38	-111.40	4.75

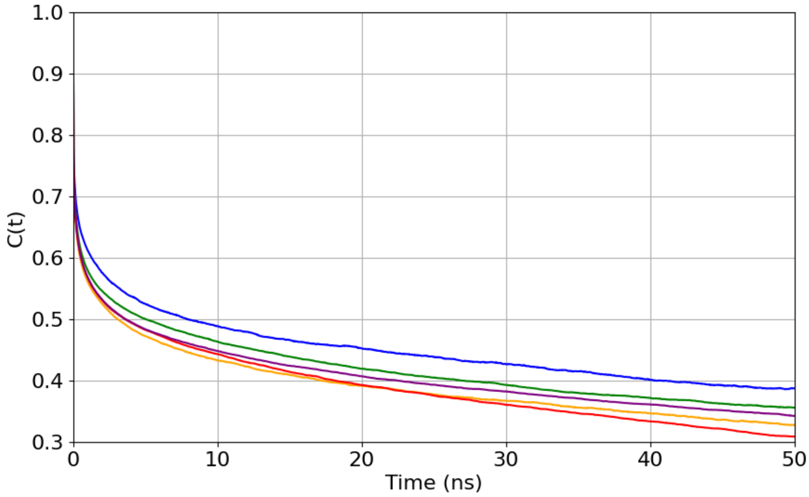

Figure S3: Autocorrelation curves (C(t)) for hydrogen bond lifetimes between peptide-peptide for different models. Model-01 = Blue; Model-02 = Yellow; Model-03 = Green; Model-04 = Red; and Model-05 = Purple.

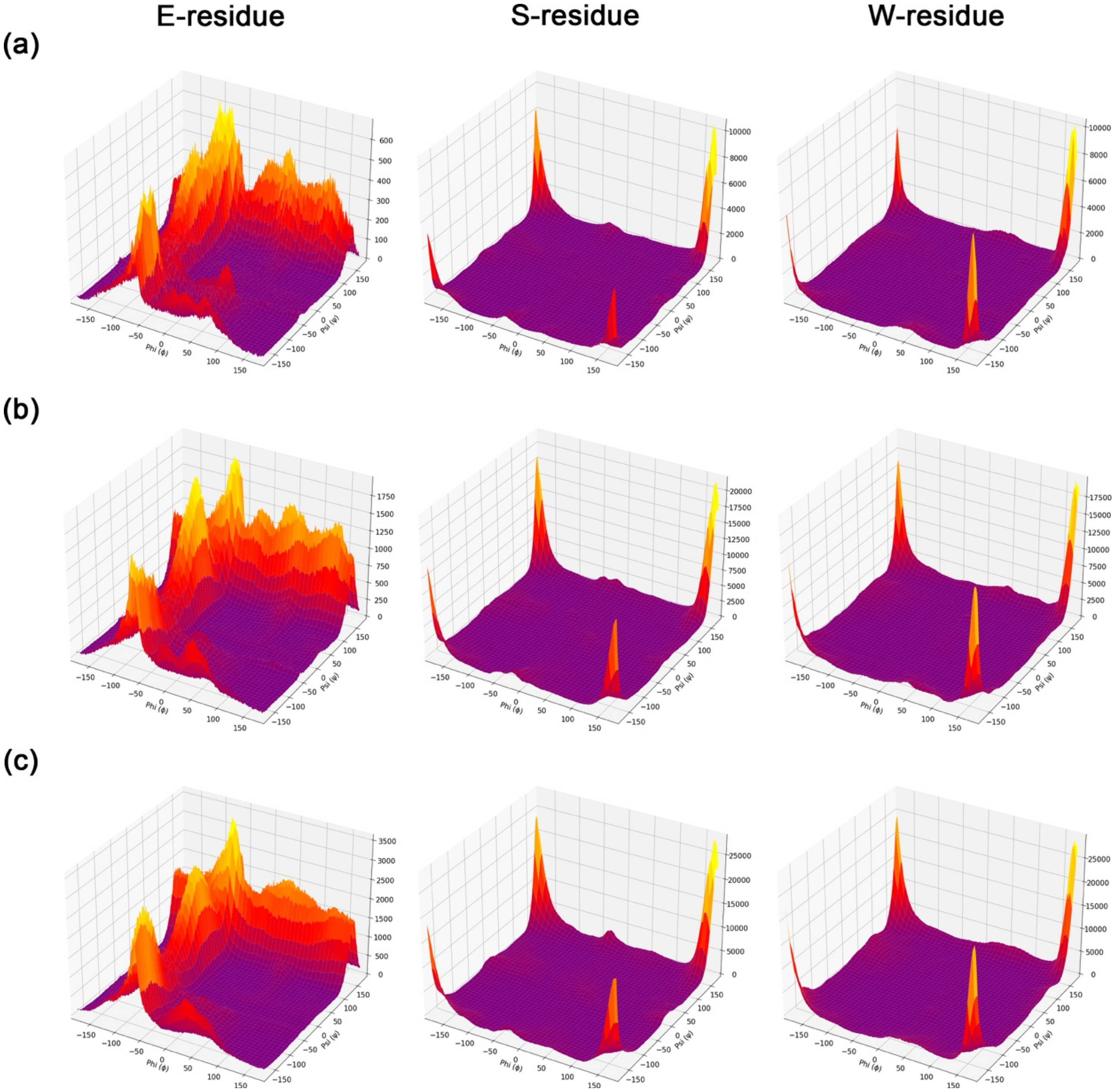

Figure S4: Ramachandran plot in 3D, highlighting the intensity of points in the preferential φ vs ψ regions shown in Figure 7. Ramachandran-3D plots highlights a few occurrences of φ vs ψ values in other regions of the plot, but these counts are considerably lower than the φ vs ψ counts that characterize the predominantly β-sheet structure. (a) Model-01; (b) Model-03; and (c) Model-05.

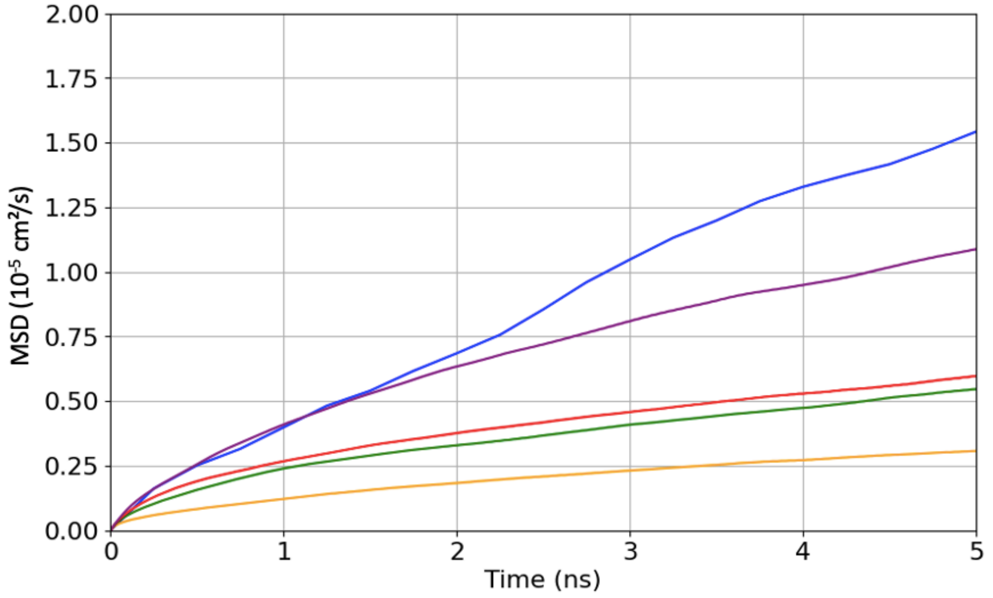

Figure S5: Mean-squared displacement curves for peptides molecules for all models. Model-01 = Blue; Model-02 = Yellow; Model-03 = Green; Model-04 = Red; and Model-05 = Purple.
